# Psychosocial risks in Latin America: trends and research gaps

**DOI:** 10.3389/fpubh.2026.1788232

**Published:** 2026-03-31

**Authors:** Sonia Raquel Vargas-Veliz, Daniel Rolando Izquierdo-Cevallos, Johanna Elizabeth Fajardo-Vargas, Damaris Kassandra Solórzano-Rezabala, Dennis Alfredo Peralta-Gamboa

**Affiliations:** 1 Universidad Estatal de Milagro, Milagro, Ecuador; 2 Universidad Tecnica Estatal de Quevedo, Quevedo, Ecuador

**Keywords:** burnout, Latin America, mental health, psychosocial risks, scientific collaboration

## Abstract

This study analyzes the scientific evolution of psychosocial risks in Latin America through a bibliometric analysis of 1,866 articles indexed in Scopus and Web of Science between 1963 and 2026. The results show sustained growth in academic production, particularly during the last decade, accompanied by an increase in impact measured by citations. The international collaboration network reveals a structure articulated in South–South and South–North patterns, with Brazil as a regional node and the United States as a transnational bridge. The keyword co-occurrence analysis identifies three main thematic clusters: (1) mental health and psychological distress—including anxiety, depression, and stress—; (2) labor and organizational factors, including burnout, working conditions, workplace violence, and job satisfaction; and (3) the disruptive effects of the COVID-19 pandemic, which reorganized the recent scientific agenda. The findings demonstrate that psychosocial risks in Latin America constitute a field in consolidation, characterized by the convergence of clinical, labor, and structural dimensions, and by growing regional visibility in the international literature. However, limitations persist, associated with the predominance of cross-sectional studies, the underrepresentation of informal and precarious sectors, and geographical asymmetries in scientific infrastructure. It is suggested to advance toward comparative, longitudinal, and interdisciplinary approaches that integrate mental health, work organization, and regional socioeconomic contexts. This study provides an empirical basis for understanding the investigative configuration of the field and guiding future research agendas, public policies, and interventions aimed at psychosocial well-being in the region.

## 1 Introduction

Psychosocial risks have gained increasing visibility in Latin America over the last two decades due to the link between working conditions, mental health, and socioeconomic transformation. Various studies have documented emotional exhaustion (1), burnout (2), workplace violence (3), and other forms of psychological deterioration, evidencing that these issues have become a structural challenge for workers and organizations. In clinical contexts, for example, recent research shows latent profiles of burnout and psychological distress among health professionals, revealing the interaction between workload, chronic stress, and well-being deterioration (4). Similarly, studies on psychological violence and burnout among Latin American physicians reinforce the idea that psychosocial exhaustion is associated with both organizational factors and structural tensions (2).

From a conceptual perspective, psychosocial risks (PSR) are not equivalent to mental health outcomes themselves, but rather refer to the set of organizational, social, and work-related conditions that increase the probability of adverse psychological, emotional, and social consequences for workers and other exposed populations. In line with integrative conceptual frameworks, PSR encompass factors such as work organization, workload, job insecurity, exposure to violence, deficient social support, and inadequate institutional regulation, which operate as upstream determinants of mental health problems, including stress, burnout, anxiety, and depression. Following the conceptual clarification proposed by Teixeira et al. (5), this study adopts a comprehensive perspective in which psychosocial risks are understood as structurally embedded exposures within work and organizational contexts, while mental health outcomes are treated as downstream manifestations of these risk configurations. This distinction allows differentiating analytically between risk-generating conditions and their psychosocial consequences, while recognizing their dynamic interrelation within broader socioeconomic and institutional environments.

Mental exhaustion is not limited to clinical professionals. In educational institutions, academic burnout syndrome is linked to anxiety, depression, and emotional burden among university students, suggesting that dynamics of pressure, precariousness, and competitiveness affect multiple social groups (6). Other works indicate that deterioration in rest and sleep quality among students and young professionals is associated with academic demands, occupational stress, and sociocultural contexts that pressure toward permanent self-demand (7). These findings reflect that the region experiences psychosocial problems that transcend specific occupations and are linked to economic structures, labor inequalities, and organizational transformations.

In parallel, research oriented toward structural labor processes indicates that dynamics such as outsourcing, informality, and territorial segmentation directly affect occupational health conditions. An analysis of the evolution of mining outsourcing in Mexico describes sectoral and social implications that reconfigure work, precarious employment ties, and influence psychosocial risks (8). This type of study reinforces the idea that psychosocial risks must be interpreted in relation to inequality, productive models, and institutional transformations—elements particularly visible in Latin America.

At the bibliometric level, the available literature evidences sustained growth in the field, but also challenges related to thematic fragmentation, geographical concentration, and limited interdisciplinary integration. Although there is abundant clinical and psychological production—such as the analysis of compassion fatigue in care settings (9)—macro-structural and comparative approaches remain incipient. This pattern reveals that the region predominantly studies symptoms and behaviors associated with distress, while greater exploration of structural risk-generating mechanisms is needed, as suggested by studies like that of Téllez-Ramírez and Sánchez-Salazar (8), which link productive and territorial conditions with labor impact.

These observations justify the value of research that integrates organizational, institutional, and structural dimensions, in addition to the individual experience of stress. Understanding psychosocial risks from a multi-scalar approach allows visualization of how macroeconomic dynamics, sociolabor precariousness, high-demand environments, and weak regulation manifest in emotional distress, organizational violence, and professional exhaustion. In this sense, bibliometric analyses offer tools to map the scientific evolution of the field and reveal patterns, emphases, gaps, and investigative opportunities.

Consequently, this study aims to analyze the evolution of research on psychosocial risks in Latin America between 1963 and 2026, examining productivity, collaboration networks, influential journals, citation patterns, and emerging thematic dynamics through a bibliometric analysis of a consolidated database of 1,866 documents.

## 2 Methodology

This study adopts a descriptive and relational bibliometric approach to analyze scientific production on psychosocial risks in Latin America between 1963 and 2026. The methodology was structured in four phases: (i) construction of the documentary corpus through systematized searches in indexed databases, (ii) data depuration and integration, (iii) analysis techniques, and (iv) interpretive synthesis of results.

The four-phase structure follows standard procedures commonly adopted in bibliometric and scientometric studies, as described in established methodological frameworks (10), including corpus construction, data cleaning and integration, analytical processing, and interpretive synthesis of results.

### 2.1 Construction of the documentary corpus

Searches were conducted in Scopus and Web of Science, selected for their global coverage, consolidated indexing, and presence of Latin American journals (11, 12). Search equations combined terms on psychosocial risks and Latin American countries were used.

The full search strings applied in each database are reported below:

Scopus: TITLE-ABS-KEY ((“psychosocial risk” OR “work stress” OR “occupational stress” OR burnout OR “job strain” OR “workplace violence” OR wellbeing OR well-being) AND (“Latin America” OR “South America” OR “Central America” OR Argentina OR Bolivia OR Brazil OR Chile OR Colombia OR Costa Rica OR Cuba OR Dominican Republic OR Ecuador OR El Salvador OR Guatemala OR Honduras OR Mexico OR Nicaragua OR Panama OR Paraguay OR Peru OR Uruguay OR Venezuela)).

Web of Science: TS = ((“psychosocial risk” OR “work stress” OR “occupational stress” OR burnout OR “job strain” OR “workplace violence” OR wellbeing OR well-being) AND (“Latin America” OR “South America” OR “Central America” OR Argentina OR Bolivia OR Brazil OR Chile OR Colombia OR Costa Rica OR Cuba OR Dominican Republic OR Ecuador OR El Salvador OR Guatemala OR Honduras OR Mexico OR Nicaragua OR Panama OR Paraguay OR Peru OR Uruguay OR Venezuela)).

In this study, psychosocial risk factors (PSR) are understood as work-related and socio-organizational conditions that increase the probability of adverse psychological or social outcomes, including stress-related disorders, burnout, and emotional distress. While a strict theoretical distinction can be made between upstream exposures (e.g., work organization, job demands, workplace violence) and downstream manifestations (e.g., burnout, diminished well-being), the Latin American literature frequently operationalizes these constructs within an integrated empirical framework. Therefore, the search strategy was designed to capture how PSR are effectively conceptualized and measured in regional research, rather than imposing a narrowly bounded theoretical definition.

The initial search detected 2,899 documents in Scopus and 1,899 in Web of Science. Subsequently, filters were applied to delimit thematic relevance: Social Sciences, Psychology, and Medicine areas; document type (scientific articles); and languages (Spanish and English). After this, 1,969 valid articles remained in Scopus and 407 in Web of Science.

The selection of these keywords is justified by their alignment with integrative conceptual frameworks of psychosocial risks (PSR), which emphasize upstream conditions such as work organization, occupational stress, and workplace violence, rather than downstream outcomes such as isolated mental disorders (5). This ensures a precise focus on the scope of the study—identifying trends and gaps in Latin America—avoiding overly broad terms (e.g., generic ‘mental health’) that would dilute relevance, while covering the clinical, occupational, and structural dimensions observed in the regional literature. The combination with country and regional names guarantees geographical focus, capturing both intraregional production and South–North collaborations.

### 2.2 Data depuration and integration

The extracted files were downloaded in.csv (Scopus) and.xlsx (WoS) formats. Integration and depuration were performed using R version 4.4.3. Data cleaning and integration were conducted in R (version 4.4.3) using the packages *dplyr*, *stringr*, and *tidyverse*. Duplicate detection was performed by standardizing titles to lowercase and applying logical matching procedures using functions such as *tolower()*, *duplicated()*, and pattern matching with *grepl()*. Manual inspection was subsequently conducted to validate ambiguous cases.

To identify duplicates, titles were standardized to lowercase and compared using logical matches with grepl(), detecting 123 duplicate records. The final corpus consisted of 2,253 documents for preliminary review of titles and abstracts, which were filtered again using textual searches on abstracts with grepl() to confirm conceptual presence of psychosocial risks and the study region. After this stage, a database of 1,866 valid documents was consolidated, constituting the final analytical sample.

Relevant variables for the study included authors, title, year, publication source, country, received citations, keywords, and abstracts. Verifications were performed to ensure consistency in years, removal of special characters, and normalization of author and institution names.

Journal source titles were standardized to address inconsistencies between databases and metadata records. This process included converting all journal names to lowercase, correcting typographical variations, harmonizing spelling and abbreviations, and merging equivalent source titles that referred to the same journal but appeared under different formats (e.g., differences in capitalization or minor orthographic variants across Scopus and Web of Science records). This standardization step was applied prior to the calculation of productivity and citation indicators by source in order to avoid artificial duplication of journals and ensure accurate attribution of publications and impact.

The selection decisions were based on explicit inclusion criteria: (i) thematic relevance, confirmed through textual searches in abstracts using grepl() to verify the presence of PSR concepts and Latin American context; (ii) alignment with the objective of identifying gaps (e.g., inclusion of studies addressing structural factors, exclusion of purely clinical cases without organizational linkage); and (iii) indexed quality (articles in the areas of Social Sciences, Psychology, and Medicine). Duplicates were excluded (123 detected through logical matching using tolower() and duplicated()), as well as non-relevant documents (387 removed after abstract review), and those in non-selected languages (Portuguese, acknowledged as a limitation). This rigorous depuration process, guided by PRIBA (13), ensures a coherent and representative corpus of 1,866 documents, prioritizing reproducibility over absolute exhaustiveness in non-indexed sources.

### 2.3 Analysis techniques

The analysis was executed in R 4.4.3 using libraries oriented toward manipulation and visualization (including dplyr, tidyverse, and native functions). Productivity was analyzed through counts of publications by year, author, and institution. Impact indicators were examined based on accumulated citations, average citations, and citation concentration in high-relevance authors and documents.

The most influential journals were identified by publication frequency and associated citations. For international collaboration analysis, affiliation and country fields were processed, generating co-authorship matrices between countries. Although R allows visual representation, in this study results are presented through solid analytical description, but the dataset allowed constructing networks where Brazil, Mexico, Chile, and Colombia appear as regional cores, while Spain, the United States, and the United Kingdom sustain external collaboration alliances.

The keyword co-occurrence analysis was built from standardized terms extracted from each record. Standardization involved reduction to lowercase, semantic unification (e.g., “violencia laboral” and “workplace violence”), and elimination of lexical duplicates. Subsequently, a pair co-occurrence matrix of keywords was established, allowing identification of semantic cores and emerging themes linked to burnout, occupational stress, institutional violence, mental health, psychosocial well-being, and workload.

In the keyword co-occurrence analysis, in addition to reporting the frequency of terms, the Total Link Strength (TLS) was considered as a relational indicator. TLS represents the sum of the strengths of all co-occurrence links of a given keyword with other keywords in the network and reflects its degree of thematic connectivity and centrality within the scientific field. Higher TLS values indicate stronger and more frequent co-occurrence relationships with other terms, suggesting greater integrative relevance in the thematic structure of the literature.

Finally, qualitative information was synthesized through content analysis of included abstracts, seeking thematic patterns, conceptual evolution, and investigative gaps.

The qualitative synthesis of abstracts followed a structured thematic content analysis approach, including open coding of recurrent concepts, grouping of codes into thematic categories (e.g., mental health, burnout, workplace violence, COVID-19-related impacts), and interpretive aggregation of patterns and research gaps. This process was conducted iteratively to identify dominant conceptual trajectories and emerging research themes within the corpus.

The analytical strategy of this study is descriptive and exploratory in nature, consistent with standard bibliometric and scientometric approaches aimed at mapping research landscapes rather than testing causal or associational hypotheses. Accordingly, no inferential statistical tests, null-hypothesis significance testing, correlation analyses, or regression models were applied at any stage of the analysis. The reported indicators (e.g., publication counts, citation metrics, co-authorship networks, and keyword co-occurrence measures) are intended to characterize structural patterns, thematic organization, and collaboration dynamics within the scientific field. Therefore, *p*-values and multiple-comparison correction procedures are not applicable to the present study.

### 2.4 Ethical considerations and reproducibility

The study is based on secondary, publicly available published data; therefore, it did not require institutional ethical approval. No sensitive personal data were used, and the analysis was oriented exclusively toward bibliographic information. To ensure reproducibility, methodological steps describe search equations, applied filters, software used, and cleaning processes. Additionally, analyses can be replicated by importing equivalent databases and repeating filtering and depuration operations in R.

In summary, this methodology allows constructing a structured, verifiable, and comparative representation of the investigative evolution on psychosocial risks in Latin America between 1963 and 2026, providing quantitative evidence to interpret the dynamics exposed in the results.

The reporting of methods and results was guided by emerging recommendations for transparency in bibliometric studies, including the Preferred Reporting Items for Bibliometric Analysis (PRIBA) framework, to enhance clarity, completeness, and reproducibility of the analytical workflow (13).

## 3 Results

### 3.1 Temporal evolution and citations

**Figure 1:**
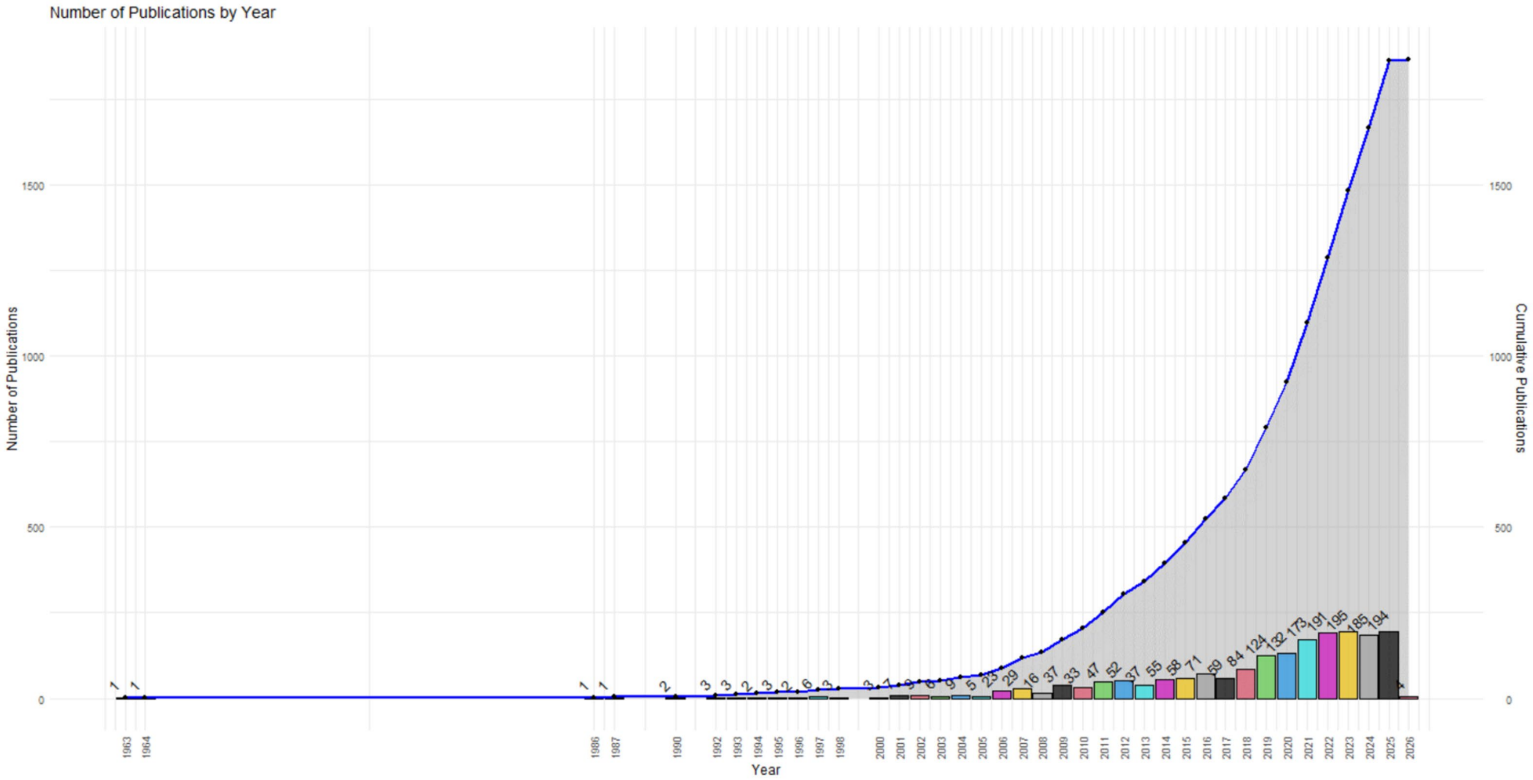
Annual and cumulative evolution of scientific publications on psychosocial risks in Latin America (1963–2026).

The analysis of the 1,866 documents included in the corpus reveals sustained growth in research on psychosocial risks in Latin America across the full period analyzed (1963–2026) (see Figure 1). In the early years, descriptive studies linked to stress and well-being predominated, while in the last decade, there was an intensification of publications oriented toward professional exhaustion, organizational violence, academic burnout, and clinical exhaustion. A marked increase in publication volume is observed from 2018 onwards, with a concentration of studies addressing burnout, workplace stress, and psychosocial risks in health and educational contexts.

**Figure 2:**
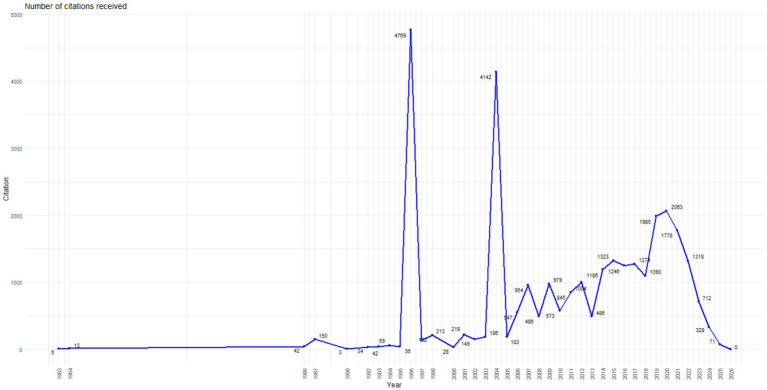
Annual evolution of citations received by scientific production on psychosocial risks in Latin America (1963–2026).

The identified production peaks between 2018 and 2024 reflect both field expansion and thematic diversification. For example, recent research has analyzed latent profiles of burnout in medical residents (4) and the relationship between psychological violence and professional exhaustion in Peruvian physicians (2). In parallel, studies on labor outsourcing and its social implications in Mexico were published (8), evidencing the incorporation of structural analyses linked to working conditions and economic production (Figure 2).

The evolution of received citations shows a significant increase in the scientific impact of the field throughout the analyzed period. Between 1963 and the late 1990s, citations were practically marginal, reflecting a formative stage of the area with low international visibility. From the 2000s decade, citation peaks associated with seminal high-influence articles are observed, followed by a phase of sustained growth between 2010 and 2021, during which the field consolidated and diversified thematically. In the most recent years (2023–2026), an expected decrease in citations is recorded due to the inherent temporal lag in citation reception for recently published articles. Overall, these results evidence a progressive increase in the scientific impact of psychosocial risks as a research area, especially during the last decade.

### 3.2 Most influential journals and publication patterns

The selected articles were published in 781 different journals. Table 1 presents the 9 journals with the highest number of published articles and their corresponding citation volume, allowing simultaneous observation of scientific productivity patterns and editorial influence in the field of psychosocial risks in Latin America.

**Table 1:** Most productive and influential journals in the scientific production on psychosocial risks in Latin America.

Source title	Quantity	Citations
International Journal of Environmental Research and Public Health	64	892
Revista Brasileira de Medicina do Trabalho	43	133
Revista de Saude Publica	26	550
Salud Mental	26	285
Frontiers in Psychology	23	668
Work	22	214
Revista Médica de Chile	40	567
Revista Colombiana de Psiquiatría	20	97
Revista de Salud Publica	18	97

In terms of productivity, the *International Journal of Environmental Research and Public Health* clearly dominates, accumulating 64 articles, suggesting strong openness of this journal to topics related to mental health, occupational health, and psychosocial factors from a public health perspective. Next, *Revista Brasileira de Medicina do Trabalho* positions itself with 43 articles, representing an important core for occupational and clinical research oriented toward work in the Latin American context. Regional journals such as *Revista de Saude Publica*, *Salud Mental*, *Revista Médica de Chile*, and *Revista Colombiana de Psiquiatría* reinforce the predominance of editorial channels specialized in public health, epidemiology, and psychiatry.

When considering impact by citations, a less linear distribution is observed. Although *International Journal of Environmental Research and Public Health* also leads in citations (892), journals like *Frontiers in Psychology* (668) and *Revista de Saude Publica* (550) show significant performance in terms of average impact, indicating that, despite lower publication volume, they concentrate highly cited articles with greater international visibility. This pattern is characteristic of psychology and health journals, where psychosocial research often has interdisciplinary audiences.

Likewise, the case of *Work*, *Salud Mental*, and *Revista Médica de Chile* suggests consistent performance in both productivity and citation, evidencing thematic capillarity across mental health, occupational medicine, and public health. It is worth noting the duplication between *Revista Medica de Chile* and *REVISTA MEDICA DE CHILE*, probably due to inconsistencies in data standardization, common in bibliometric databases.

### 3.3 International collaboration networks and research geography

**Figure 3:**
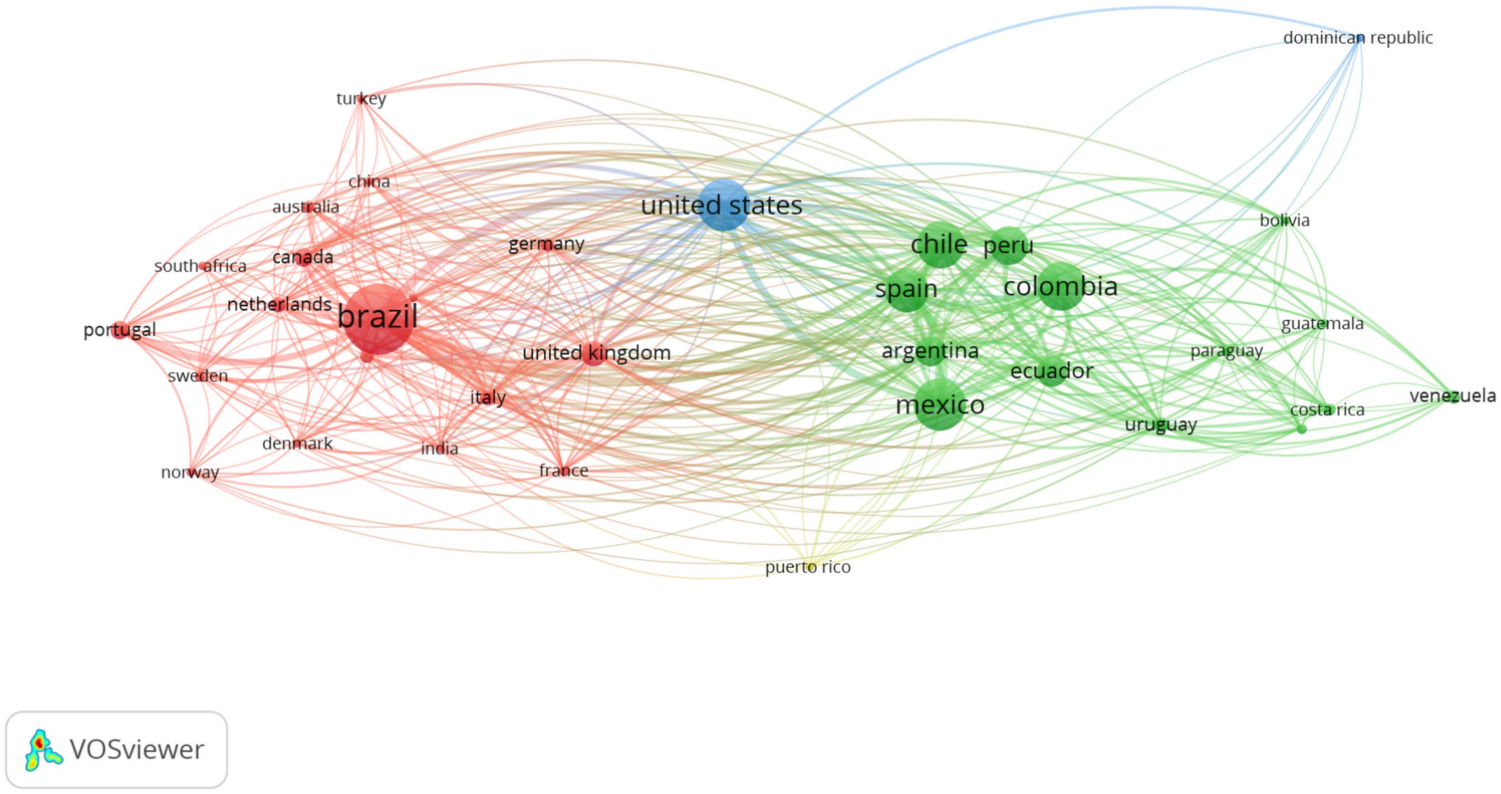
International co-authorship network in scientific production on psychosocial risks in Latin America.

The international co-authorship network revealed three main clusters (see Figure 3). The first, led by Brazil, is primarily articulated with European countries (United Kingdom, Netherlands, Italy, France, and Portugal). The second corresponds to a Latin American core integrated by Chile, Peru, Colombia, Mexico, Argentina, Ecuador, Uruguay, and Venezuela, evidencing intraregional collaboration in scientific production. The third cluster is headed by the United States and links both the Latin American and European cores, acting as a bridge node between them. These results indicate that Latin American research on psychosocial risks is structured through South–South and South–North collaboration networks, with Brazil as a regional productivity center and the United States as an articulator of international connections.

### 3.4 Keyword co-occurrence and thematic evolution

The keyword co-occurrence analysis revealed a field organized into three main thematic clusters. The first corresponds to the clinical-psychosocial axis (37, 38, 42, 46), with terms such as *mental health* (170 occurrences; 481 TLS) (40, 41), *depression* (115; 327) (39), and *stress* (95; 245).

**Figure 4:**
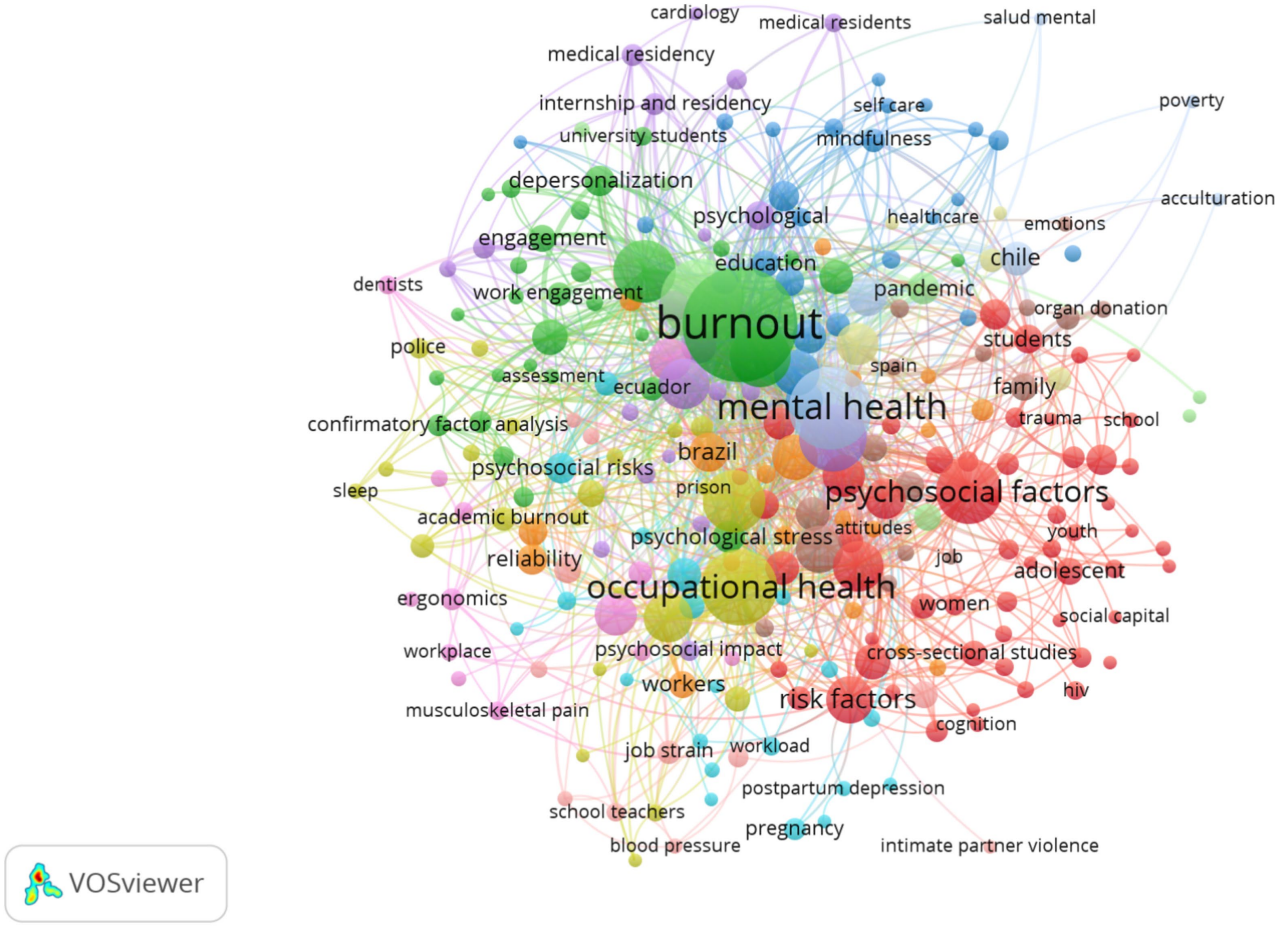
Thematic keyword co-occurrence network of psychosocial risks research in Latin America.

In this context, the Total Link Strength (TLS) is used to indicate the overall intensity of co-occurrence relationships of each keyword within the thematic network. Thus, keywords with high TLS values not only appear frequently but also maintain strong relational links with multiple thematic nodes, reflecting their structural centrality in the organization of the research field (Figure 4).

The second cluster is oriented toward the labor-organizational dimension, where *burnout* stands out as a central term (332; 773), along with *occupational health* (146; 397), *occupational stress* (97; 194), *working conditions* (62; 173), and *workplace violence* (57; 113) (43). The third cluster groups terms associated with the COVID-19 pandemic (*covid-19*, 163; 453), evidencing the disruptive impact of this health event on recent production. The presence of the descriptor *Latin America* (44; 95) confirms the recognition of the region as an analytical unit in the scientific literature. Overall, the thematic structure indicates articulation between mental health, labor risk, and global events, with burnout as the dominant category in the field.

### 3.5 Influential studies and qualitative synthesis of the literature

To complement the quantitative bibliometric indicators, a qualitative synthesis of the most influential studies in the field was conducted. The selection considered highly cited publications and studies that contributed conceptually or empirically to understanding psychosocial risks in Latin America.

Table 2 summarizes representative influential articles, indicating their country of study, methodological approach, and main findings. This synthesis allows bridging bibliometric patterns with substantive scientific contributions, providing contextual interpretation of the thematic structure identified in the keyword analysis and citation dynamics.

**Table 2:** Influential studies on psychosocial risks in Latin America.

Author	Country	Methodology	Main findings
Lozano et al. (1)	Global	Systematic review	Identified workplace violence and burnout as key psychosocial risk factors among healthcare workers.
Flores-Cohaila et al. (4)	Peru	Quantitative (latent profile analysis)	Revealed different burnout profiles among medical residents associated with workload and emotional exhaustion.
Nombera-Aznaran et al. (2)	Peru	Cross-sectional quantitative	Demonstrated the relationship between psychological workplace violence and burnout among physicians.
Menacho-Rivera et al. (6)	Peru	Multivariable regression	Found strong associations between academic burnout, anxiety, and stress among university students.
Chauca-Bajaña et al. (16)	Ecuador	Multicenter cross-sectional	Reported high prevalence of burnout among dental students in clinical training contexts.
Pereira dos Santos et al. (30)	Brazil	Quantitative survey	Identified occupational stress patterns among nursing professionals in public hospitals.
Arango-Lasprilla et al. (20)	Global	Quantitative survey	Highlighted predictors of emotional exhaustion among teachers after COVID-19.
Téllez-Ramírez and Sánchez-Salazar (8)	Mexico	Structural labor analysis	Linked outsourcing processes with changing labor conditions and psychosocial risk exposure.

The reviewed studies show that research in the region has focused primarily on occupational groups exposed to high emotional demands, particularly health professionals, teachers, and university students. Cross-sectional quantitative designs predominate, frequently using psychometric instruments to measure burnout, stress, and psychosocial distress. At the same time, qualitative and mixed-method studies provide contextual insights into organizational violence, institutional pressures, and sociocultural determinants of mental health.

Overall, these studies confirm that psychosocial risks in Latin America are shaped by the interaction between organizational conditions, socioeconomic inequalities, and institutional environments, reinforcing the need for integrated research approaches that combine occupational health, psychology, and social policy perspectives.

## 4 Discussion

The results of this study indicate that psychosocial risks have consolidated as an expanding research field in Latin America, driven by sustained growth in scientific productivity, increasing citation impact, and thematic diversification over the last two decades. This expansion is reflected in the diversification of studied populations and analytical contexts, encompassing health professionals and medical residents (4, 14, 15), university students (6, 16), and teachers (17, 18), among other occupational groups exposed to high emotional and organizational demands (36). Collectively, these trends suggest a gradual transition from predominantly clinical and descriptive approaches toward a broader research agenda that integrates labor, educational, and community dimensions of psychosocial risk. The qualitative synthesis presented in Table 2 helps contextualize the bibliometric patterns identified in the Results section, showing how highly cited studies have contributed to understanding psychosocial risks across different occupational groups and institutional contexts in Latin America.

This shift can be partly explained by several converging factors. First, the expansion of occupational health and public health frameworks in Latin America has encouraged researchers to situate psychosocial risks within broader organizational and institutional contexts rather than treating them solely as individual clinical outcomes. Second, increasing recognition of labor precariousness, informality, and structural inequalities in the region has fostered interest in work-related and community-level determinants of psychosocial distress. Third, policy agendas and international guidelines on decent work, mental health promotion, and workplace well-being have stimulated interdisciplinary research agendas that bridge psychology, sociology, public health, and labor studies. Together, these dynamics help explain the observed reorientation of the field toward more contextualized and socially embedded perspectives on psychosocial risks.

In terms of scientific impact, the concentration of recent publications addressing emotional exhaustion, depersonalization, and psychological distress across diverse occupational groups aligns with the observed increase in citation dynamics, particularly after 2018. The COVID-19 pandemic appears to have acted as a critical inflection point, intensifying scholarly attention to psychosocial factors at work and accelerating research output. Empirical evidence from multiple countries indicates that prolonged exposure to labor demands, uncertainty, care overload, and organizational disruption during and after the pandemic translated into elevated risks of stress, burnout, and deterioration of well-being (19–26). This pattern reinforces the role of large-scale socio-health crises as catalysts that reorganize research priorities and reshape the thematic configuration of the field.

The international co-authorship network further indicates that Latin American research on psychosocial risks is embedded within global scientific collaboration circuits rather than operating in isolation. Collaborative studies addressing mindfulness- and compassion-based interventions (27), burnout risk and organizational stress among physicians and residents (28, 29), and psychosocial experiences of teachers and health professionals (30) demonstrate sustained cooperation among institutions from Peru, Colombia, Chile, Brazil, Mexico, and extra-regional partners. While asymmetries in research capacity and international visibility persist across countries, these collaboration patterns highlight the region’s active participation in global debates on mental health and psychosocial risks, alongside the production of contextually grounded evidence reflecting local labor and social realities.

Rather than reiterating the thematic axes identified in the Results section, these patterns can be interpreted as reflecting the structural configuration of psychosocial risk research in Latin America. The prominence of clinically oriented themes is consistent with the historical dominance of mental health research in the region, where psychosocial risks have traditionally been operationalized through indicators of distress, burnout, and emotional suffering. The salience of labor- and organization-centered themes reflects the centrality of work as a site of psychosocial vulnerability in Latin America, characterized by high levels of informality, job insecurity, exposure to workplace violence, and limited institutional protection. Finally, the strong visibility of COVID-19-related topics can be understood as a consequence of the pandemic’s disruptive impact on working conditions, care systems, and mental health, which rapidly reoriented research agendas toward crisis-related psychosocial vulnerabilities. Together, these thematic concentrations suggest that the field is shaped by the intersection of long-standing clinical traditions, structurally embedded labor conditions, and acute socio-health shocks.

In the Latin American context, the centrality of categories such as burnout, mental health, and occupation reflects that psychosocial risks cannot be understood solely as clinical symptoms, but as manifestations of broader sociohistorical processes involving productive transformations, restructuring of care systems, and persistent tensions between institutional expectations and available resources. In this sense, the thematic configuration of the field mirrors structural features of the region, where labor precariousness, unequal access to care, and institutional fragility shape both exposure to psychosocial risks and the scholarly agendas that seek to address them.

An additional contribution of this study is the identification of Latin America as an explicit analytical unit within the scientific literature, rather than merely a collection of isolated national case studies. Multicenter and regionally framed research increasingly recognizes the specific structural conditions of the region—such as labor informality, social inequality, and institutional fragility—as contextual determinants shaping exposure to psychosocial risks and patterns of emotional distress (4, 6, 14, 20). This emerging regional framing supports the development of comparative and theoretically grounded approaches capable of capturing both shared vulnerabilities and cross-country heterogeneity within Latin America.

The increasing visibility of “Latin America” as a descriptor in thematic analyses suggests that the region is progressively being recognized as an analytical unit rather than merely as a set of isolated national cases, opening opportunities for the development of comparative research agendas and regionally grounded interpretive frameworks.

Despite the observed consolidation of the field, several structural gaps remain. The predominance of cross-sectional designs, the concentration on specific occupational groups (particularly health professionals, teachers, and students), and the limited attention to informal, rural, and highly precarious labor sectors point to partial coverage of psychosocial risks in the region (16, 28, 30–32). Moreover, intervention-oriented research, preventive strategies, and community-based psychosocial programs remain comparatively underdeveloped relative to the volume of diagnostic and prevalence studies. Although emerging evidence points to promising experiences in mindfulness-based interventions, compassion-focused approaches, programs addressing workplace violence, and suicide prevention among young populations (29, 33–35, 44, 45), these lines of inquiry remain fragmented and require stronger longitudinal and evaluative designs. Overall, the findings portray a dynamic and consolidating research field that would benefit from the development of comparative, longitudinal, and interdisciplinary frameworks capable of jointly articulating clinical, organizational, and structural dimensions of psychosocial risks in Latin America.

### 4.1 Limitations

The limitations of this study derive primarily from the nature of the bibliometric approach and the characteristics of the selected documentary corpus.

First, although Scopus and Web of Science provide extensive international coverage and are widely recognized for their indexing rigor (11), reliance on these databases may exclude relevant publications available in non-indexed regional journals, institutional repositories, or alternative databases. This may result in the partial underrepresentation of certain countries or locally oriented research traditions within Latin America.

Second, the restriction of the corpus to publications in English and Spanish implies the exclusion of studies published in Portuguese. Given Brazil’s prominent role in psychosocial risk research in the region, this language limitation may have led to the underrepresentation of Brazilian scholarship, particularly older studies disseminated through national journals or outlets not indexed in the selected databases. Consequently, some historical trajectories and contextually grounded research traditions may not be fully captured in the present analysis.

Third, the descriptive and exploratory nature of bibliometric analysis does not allow for the assessment of methodological rigor, instrument validity, or internal quality of the included empirical studies. Nor does it permit causal inference or statistical testing of associations between variables such as thematic evolution, collaboration intensity, or citation impact. The findings should therefore be interpreted as structural patterns of scientific production rather than as statistically validated relationships or evidence of causal dynamics within the field.

Additionally, the operational breadth of the search terms may have influenced the configuration of the resulting corpus. Because the Latin American literature frequently integrates psychosocial risk exposures and related outcomes (e.g., burnout or well-being) within shared analytical frameworks, the inclusion of such descriptors may have expanded the scope of retrieved studies beyond strictly defined upstream risk factors. While this decision allowed a broader representation of how the construct is empirically operationalized in the region, it may also have contributed to thematic heterogeneity within the dataset.

Finally, although the study sought to ensure reproducibility and transparency in corpus construction, the predominance of cross-sectional designs in the reviewed literature reflects a structural limitation of the field itself. The scarcity of longitudinal and intervention-based research constrains the interpretive depth of the available evidence and highlights the need for more robust methodological diversification in future studies.

Future research may benefit from expanding database coverage (e.g., inclusion of regional indexing systems), incorporating Portuguese-language production more systematically, and complementing bibliometric mapping with qualitative or systematic review approaches aimed at assessing methodological quality and theoretical coherence.

### 4.2 Future research areas

From the results, several future research directions can be outlined. Longitudinal designs are needed to analyze how sustained exposure to psychosocial risk factors is related to longitudinal trajectories of psychosocial outcomes (e.g., exhaustion, burnout, and psychological distress) across different occupational and social contexts. Such approaches would allow moving beyond cross-sectional snapshots and contribute to a more dynamic understanding of how psychosocial risks operate over time in Latin American settings.

From a comparative perspective, it would be pertinent to analyze differences between countries and subregions of Latin America to identify how structural variables—such as labor protection, health governance, or educational models—modulate risk exposure. Finally, the field could benefit from interdisciplinary approaches integrating psychology, sociology, public health, labor economics, and social policies, as well as studies oriented toward validating instruments and developing culturally contextualized interventions.

### 4.3 Practical and policy implications

The results of this study have relevant implications for the design of public policies, preventive programs, and institutional strategies oriented toward promoting psychosocial well-being. The strong presence of burnout, occupational stress, and emotional deterioration among care professionals and the educational sector suggests the need to strengthen occupational health policies, well-being monitoring, training in emotional regulation, and reporting mechanisms for organizational violence. In the educational field, the findings can inform interventions oriented toward students and teachers, including psychological support, tutoring, curricular redesign, and strategies to mitigate overload and academic anxiety.

At the macro level, the integration of Latin America as an analytical unit evidences that institutional responses must consider the structural particularities of the region—such as labor informality, social inequality, and health fragility—requiring policies that combine mental health, employment, and social cohesion. In this sense, psychosocial risks should not be treated solely as clinical phenomena but as public problems intersecting labor, economic, and community dimensions.

## 5 Conclusion

This study shows that psychosocial risks constitute a consolidating research field in Latin America, characterized by sustained growth in scientific productivity, an increase in impact measured by citations, and thematic diversification influenced by clinical, labor, and structural dynamics. Mental health, burnout, and occupational factors emerged as central conceptual cores, while the COVID-19 pandemic acted as a turning point that accelerated academic production and reconfigured analytical priorities. Likewise, collaboration networks evidenced active insertion of the field into international cooperation circuits, with Brazil as a regional node and the United States as a transnational bridge.

The findings confirm that Latin American research on psychosocial risks is in a transition phase toward greater scientific maturity, driven by the incorporation of diverse populations, explicit recognition of the region as an analytical unit, and articulation between clinical, labor, and social dimensions of distress. However, the field maintains tensions and challenges that condition its future development.
